# C - Reactive Protein Levels in Patients with Periodontal Disease and Normal Subjects

**Published:** 2013

**Authors:** Mahdieh Shojaee, Majid Fereydooni Golpasha, Ghorban Maliji, Ali Bijani, Seyed Mohsen Aghajanpour Mir, Seyede Narges Mousavi Kani

**Affiliations:** 1 *School of Dentistry, Babol Universityof Medical Sciences, Babol, Iran.*; 2 *Dental Material Reaserch Center, Assistant Professor of the Periodentology Department, School of Dentistry, Babol University of Medical Sciences, Iran.*; 3 *Cellular and Molecular Biology Research Center (CMBRC), Babol Universityof Medical Sciences, Babol, Iran.*; 4 *Department of Immunology, School of Medicine, Babol University of Medical Sciences, Iran.*; 5 *Non-Communicable Pediatric Disease Research Center, Babol University of Medical Sciences, Babol, Iran.*

**Keywords:** CRP, periodontitis, gingivitis, saliva

## Abstract

Although periodontitis is a chronic inflammatory disease but some factors of acute inflammation phase are involved in this disease among which is the C-Reactive protein (CRP). To minimize its effects, anti-inflammatory drugs or non-pharmacological approaches such as oral hygiene is recommended. CRP can also be used for the prediction and early detection of periodontal disease. The aim of the present study was the comparison of the amount of salivary C-Reactive protein (CRP) in healthy subjects and patients with periodontal disease. This case-control study was done on 90 patients referred to the Department of Periodontology of Babol Dentistry School. These subjects were divided into three groups of healthy (n = 30), gingivitis (n = 30), and chronic periodontitis (n = 30), based on Gingival Index (GI) and Clinical Attachment Loss (CAL) indices. 2ml saliva samples were collected from these people and clinical indicators including GI, CAL, Periodontal Pocket Depth (PPD), and Bleeding Index (BI) were assessed. ELISA method was used to evaluate the salivary CRP levels. Collected data were analyzed using SPSS statistical software by non-Parametric Kruskal-Wallis and Mann-Whitney test and Spearman correlation coefficient and P<0.05 was considered significant. The mean salivary CRP levels were 5332.62±5051.63pg/ml in periodontitis patients, 3545.41±3061.38pg/ml in gingivitis group and 3108.51±3574.47pg/ml in healthy subjects. The statistic analysis showed a significant difference in salivary CRP concentrations between the periodontitis patients and healthy subjects (P=0.045). The results indicate that there is a significant association between periodontitis and salivary CRP concentrations.

## Introduction

Periodontitis is an inflammatory disease of the supporting tissues of the teeth which is caused by specific microorganisms and characterized by extensive destruction of periodontal ligament and alveolar bone with pocket formation, gingival recession or both. Gingivitis is a gum inflammatory disease and clinically the presence of identifiable attachment loss in periodontitis makes it be distinguishable from gingivitis (1).

Host immune response to pathogens such as bacteria in the teeth plaque biofilm is as cooperation of innate and acquired immune system. Although periodontitis is a chronic inflammatory disease but the agents of the acute phase of inflammation which belong to the innate immune system are involved in the disease. These gents may cause the activation of complement system, neutralization of pathogenesis agents, stimulation of repair systems and degeneration of different tissues. C - reactive protein (CRP), plasminogen-activator inhibitor 1 (PAI-1) and fibrinogens are the most important factors in the acute phase (2). CRP is protein synthesized in the liver and the major protein of plasma. Its half-life is approximately 6-4 hours. The serum levels of this protein increase rapidly within 24 to 72 hours in conditions of inflammation or tissue damage and will subside after the removal of inflammation or infection (3).

CRP has given much attention due to its key role in atherosclerosis. So, that if it increases by more than 0.5 milligrams per liter, the risk of cardiovascular diseases increases (2). Several studies have also suggested a correlation between periodontitis and cardiovascular diseases. In some studies, it has even been claimed that there are some correlations between periodontal disease and atherosclerotic heart disease and heart attacks and strokes (2). Although their cause and effect relationship has not been established, but it is likely that elevation of CRP levels in periodontitis may help to understand the relationship between cardiovascular diseases and periodontitis (2).

Several investigations regarding the relationship between salivary CRP levels and periodontal disease have also been done. Among these researches, one can refer to the study of Giannobile et al. in 2009 (4) who showed that the saliva and serum CRP levels were elevated in patients with chronic and aggressive periodontitis. The purpose of this study was to evaluate salivary CRP levels, clinical attachment loss (CAL), gingival index (GI) and periodontal pocket depth (PPD) indices and the correlation between these indices and CRP saliva levels.

## Materials and Methods

**Patients**

In this case-control study, the patients referred to the department of periodontology and oral disease diagnosis of Babol Dental School who fulfilled the following requisites were studied.

Lack of asystemic disease, not taking antibiotics over the last month, no intra oral lesions, not doing scaling and root planning not doing periodontal surgery at least within the last 6 months, taking no medication that would affect the periodontal tissues and not being an active smoker. The number of people required for this study was based on a confidence level of 95% α= 05/0, the power =80% and σ1=5 and σ2=3 and d=3 was calculated as 30 subjects for each group.

These subjects were categorized based on gingival Index, clinical attachment loss indices into 3 equal groups of healthy, plaque-induced gingivitis and chronic periodontitis. The control group consisted of 30 patients with GI = 0, CAL = 0. The gingivitis group included 30 patients with GI ≥ 1 and CAL = 0. The chronic periodontitis group included 30 patients with GI ≥ 1, CAL> 2 (moderate to severe chronic periodontitis(.The subjects who participated in the study were matched in terms of age and gender.

**Saliva CRP analysis**

Firstly, by using spitting method, unstimulated saliva sample was collected from each subject. All patients were asked to avoid eating, drinking, chewing gum, and brushing an hour before collecting samples. They were given sterile capped-tubes and were asked to put 2 mm of their saliva sample into those tubes. Then an evaluation of clinical criteria (CAL), (GI), (PPD) was done. Barnett method was used to calculate bleeding index. After samples were collected, they were transported to the laboratory and kept under -80 ^0^C until the day of experiment. The ELISA method (Salimetric kit, USA, code number 1-3302) was used to evaluate salivary CRP level.

**Statistical analysis**

The collected data were analyzed using statistical software SPSS Version 20 using non-Parametric Kruskal-Wallis and Mann-Whitney test due to abnormal distribution and Spearman correlation coefficient was analyzed. P<0.05 was considered significant.

## Results

In this study, 30 subjects (12 males and 18 females ) in chronic periodontitis group with mean age of 39.73 years ±5.31, 30 subjects (15 males and 15 females) in gingivitis group with mean age of 35.07 years ±5.62, and 30 healthy volunteers (13 males and 17 females) with mean age of 34.6 years ±6.61 as the control group were evaluated.

**Fig. 1:**
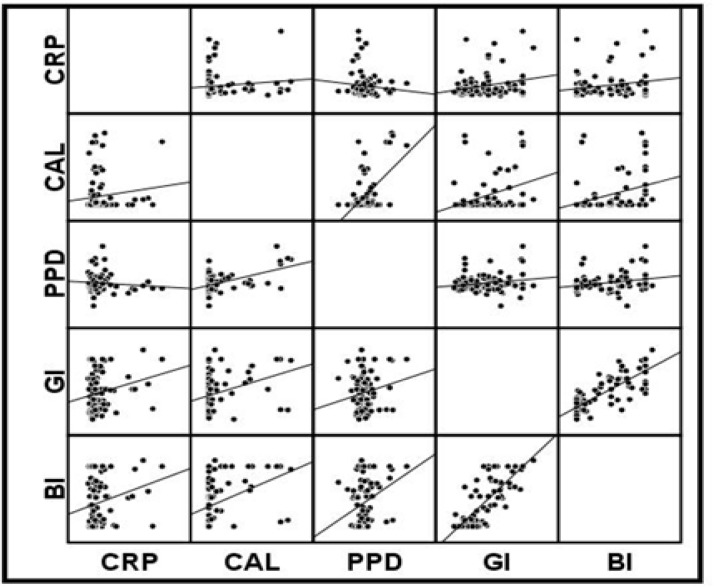
The correlation between saliva CRP levels and periodontal indices

**Fig. 2:**
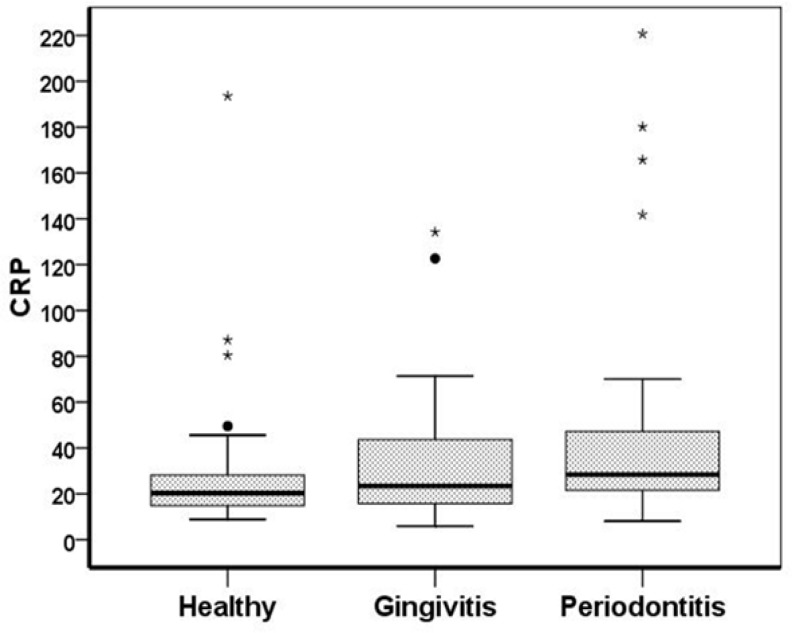
Distribution and concentration of the salivery CRP

Salivary CRP levels in chronic periodontitis group were the highest and CRP levels in healthy subjects were the lowest among the three groups. As shown in table 1, saliva CRP levels between the three groups including: pantients with periodontitis, gingivitis and control group, were statistically different (P= 0.045 .(However using Mann-Whitney test, statistically significant result for CRP level was obtained only when the perio-dontitis group was compared with the control group (P=0.01) (Fig. 1). Also, the statistical analysis showed positive correlation between saliva CRP levels and periodontal indices, including CAL, BI, GI (Fig. 2).

## Discussion

The results of the present study showed that salivary CRP concentrations increase in patients with periodontitis comparing to gingivitis and healthy subjects, confirming this theory that salivary CRP is increased in inflammatory conditions.

In studies done by Pitiphat et al. 2008 (5), Wohlfeil et al. 2011 (6) and Haba et al. 2011 (7), an increase of serum CRP levels were approved in patients with periodontitis. Our study also shows an increase of the amount of saliva CRP in patients with periodontitis. However, some patients of gingivitis group showed the numerical values close to periodontitis group which can be due to the proximity of change of the gingivitis lesion to chronic periodontitis lesion which was not clinically detectable. Also, in some of subjects of the healthy group, salivary CRP concentrations were close to the gingivitis group.

Kamil et al. in 2011 (8) investigated the effects of treatment of advanced periodontitis on serum CRP levels and the results showed that the serum CRP level decreased significantly after the non-surgical treatment, and the decrease in CRP had a significant relationship with decrease in PI, BI, GI in a direct and linear way .After statistical analysis, it was observed that the periodontitis patients had the highest CRP levels followed in order by gingivitis patients and healthy subjects. These results suggest that periodontal problems can affect the salivary CRP level and enhance it. Also, in the present study, an increase in CRP levels had a direct and significant relationship with increasing values of GI BI, and CAL. This finding also approves the findings of Kamil et al. (8).

AL-Zahrani et al. in 2012 (9) evaluated the effects of periodontitis on serum CRP levels. Their data showed that CRP levels and periodontal health indices (PI, BI, PPD, CAL) decreased significantly after treatment. Kanaparthy et al. (10). showed that CRP serum levels in patients with aggressive periodontitis were significantly higher than the control group. This study also suggests the relationship between saliva CRP and periodontitis. Pitiphat et al. in 2008 (5) investigated the association between serum CRP levels and periodontitis. The results showed that CRP levels were higher in groups of generalized periodontitis and localized periodontitis than in healthy individuals and their results are consistent with the findings of the present study.

 In the study of Pitiphat et al. (5), after adjusting for factors such as smoking and age, CRP level was significantly higher in the patients than in the healthy group. These findings suggest that smoking has important effects on the various components of the body's immune and inflamma-tory system and affects the pathogenesis and treatment outcome of periodontal disease. However, the requirement for inclusion of patients in our study were being non-smoker and matched with other groups according to age. Azar et al. in 2011 (11) compared CRP levels in the saliva of smokers and non-smokers. The results showed a considerable increase in salivary CRP concentra-tions in smokers compared to non-smokers. These results confirmed the study of Pitiphat et al. (5) who found that after eliminating smokers from the study, serum CRP levels decreased. This finding represents the beginning phases of inflammation in the body due to smoking. The mechanism of inflammation is not completely understood but probably the chemical absorption of nicotine and the effects of reactive oxygen species in cigarette, in turn cause attraction and activation of neutrophils (12). In 2009, Giannobile et al. (4) examined the serum and salivary levels of CRP and the results showed that CRP levels in serum and saliva of patients with periodontitis and patients with chronic and progressive periodontitis have increased. 

Our study showed that CRP levels also increased in parallel to the severity of periodontal diseases. It seems that this similar finding is because of the presence of inflammatory phase in periodontal disease.

Afrah et al. in 2013 (13) examined the salivary CRP levels in diabetic and non-diabetic patients with periodontitis. They showed that salivary CRP levels in the control group was lower than the other two groups and there was no significant diff-erences between the diabetic and non diabetic patients with periodontitis. The results of this study indicate that diabetes as a metabolic disease, had no effect on salivary CRP levels but the periodontitis due to its inflammatory nature, increases salivary CRP. The results of this study are also in agreement with our findings and others.

**Table 1:** CRP Concentrations mean

**P-value**	**CRP Mean± SD(pg/ml)**	**Number**	**Group**
0.045	3108.51±3574.47	30	Healthy
0.045	3545.41±3061.38	30	Gingivitis
0.045	5051.63±5332.62	30	Periodontitis

According to our data, there is a salivary CRP concentration difference between the healthy subjects and patients with gingivitis, also the difference in concentration between patients with gingivitis and periodontitis was not significant. However, based on further analysis of the samples, providing a larger sample size, these differences might be significant.

Our study is one of the few studies which compared salivary CRP concentrations in healthy subjects and patients with gingivitis and periodontitis. Based on the findings of our study and the above mentioned studies, it seems that the relationship between periodontal disease and salivery CRP is the same as their relationship with serum CRP. The results of this study shows a significant correlation between salivary CRP levels and severity of periodontal disease. Our study showed that the measurement of salivary CRP can be used as a non-invasive and reliable test for the detection and screening of periodontal disease in healthy people.
